# Complex Closed Spinal Dysraphism Presenting As Cauda Equina Syndrome With Faun Tail Nevus

**DOI:** 10.7759/cureus.47396

**Published:** 2023-10-20

**Authors:** Kashish Khurana, Shilpa A Gaidhane, Sourya Acharya, Neha Shetty

**Affiliations:** 1 Department of Medicine, Jawaharlal Nehru Medical College, Datta Meghe Institute of Higher Education and Research, Wardha, IND; 2 Department Of Medicine, Jawaharlal Nehru Medical College, Datta Meghe Institute of Higher Education and Research, Wardha, IND; 3 Department of Radiology, Jawaharlal Nehru Medical College, Datta Meghe Institute of Higher Education and Research, Wardha, IND

**Keywords:** tethered cord syndrome, cauda equina syndrome, diastematomyelia, spina bifida occulta, faun tail nevus

## Abstract

A posterior midline cutaneous lesion known as a faun tail nevus or aberrant lumbar hypertrichosis is significant to doctors because it may serve as a cutaneous signal for an underlying spinal cord and spine abnormalities. We describe a 17-year-old child who, since infancy, has had excessive hair development over his lumbosacral area. The lower spinal cord was affected by a related spinal abnormality. Clinical evidence was used to make the diagnosis. The patient presented with complaints of asymmetric monoparesis which on clinical examination and radiological investigations was found to be cauda equina syndrome secondary to spina bifida occulta. This case is reported for its clinical importance as patients with spina bifida occulta may show late deterioration.

## Introduction

A rare but deadly neurological disorder known as cauda equina syndrome (CES) affects the group of nerve roots at the base of the spinal cord. The cauda equina is composed of the caudal nerve roots below the first lumbar root, which are where the spinal cord terminates around L1. The roots descend practically vertically to the foramina that correspond to them, which are clustered around the filum terminale inside the spinal theca. The lower limbs are innervated by the cauda equina, which also innervates sphincters that control the function of the bladder and distal colon [1].

Spinal dysraphisms (SDs), congenital anomalies of the spinal cord, are caused by disruption in the intricate series of embryologic processes involved in spinal development [2]. Spinal dysraphism is a term used to describe a variety of disorders involving abnormalities in the development of the spine and spinal cord, and it occurs between 3.2 and 4.6 times per 10,000 infants [1]. 

Here, we present a case of complex closed spinal dysraphism presenting as cauda equina syndrome with abnormal lumbar hypertrichosis with diastematomyelia and tethered cord syndrome. We will also discuss the embryology of SDs.

## Case presentation

A 17-year-old boy presented to the Acharya Vinoba Bhave Rural Hospital (AVBRH) with complaints of weakness in the right lower limb with pain and tingling sensation in the right lower limb for two to three years. He developed weakness in right lower limb which was gradual in onset and progressive in nature such that earlier patient was able to walk for long distances (2-3 km) and play contact sports but for the last two to three years he has been unable to walk for more than 500 metres and cannot play any sports limited by weakness in the right lower limb. It was also associated with gait disturbance such that the right leg would go limp while walking. He also complained of slippage of chappal while walking.

In the last two to three years patient also had complaints of pain and tingling sensation in the right lower limb which starts from the lower back sacral region and radiates along the buttock, back of thigh, and leg extending to the foot. It was perceived after doing more than ordinary activities and after prolonged sitting and were relieved in lying position.

He also complained of decreased sensations over his right lower leg in the form that he is not able to feel his clothes. He also noticed diminished sensation of hot and cold temperature while bathing. He had no history of bowel and bladder involvement, abnormal body movements, swallowing difficulty, or slurring of speech. He also gave a history of tuft hair in the lower back and thinning of the right lower limb.

He was delivered as a full-term normal vaginal home delivery, without undue prolongation or complication.

**Figure 1:**
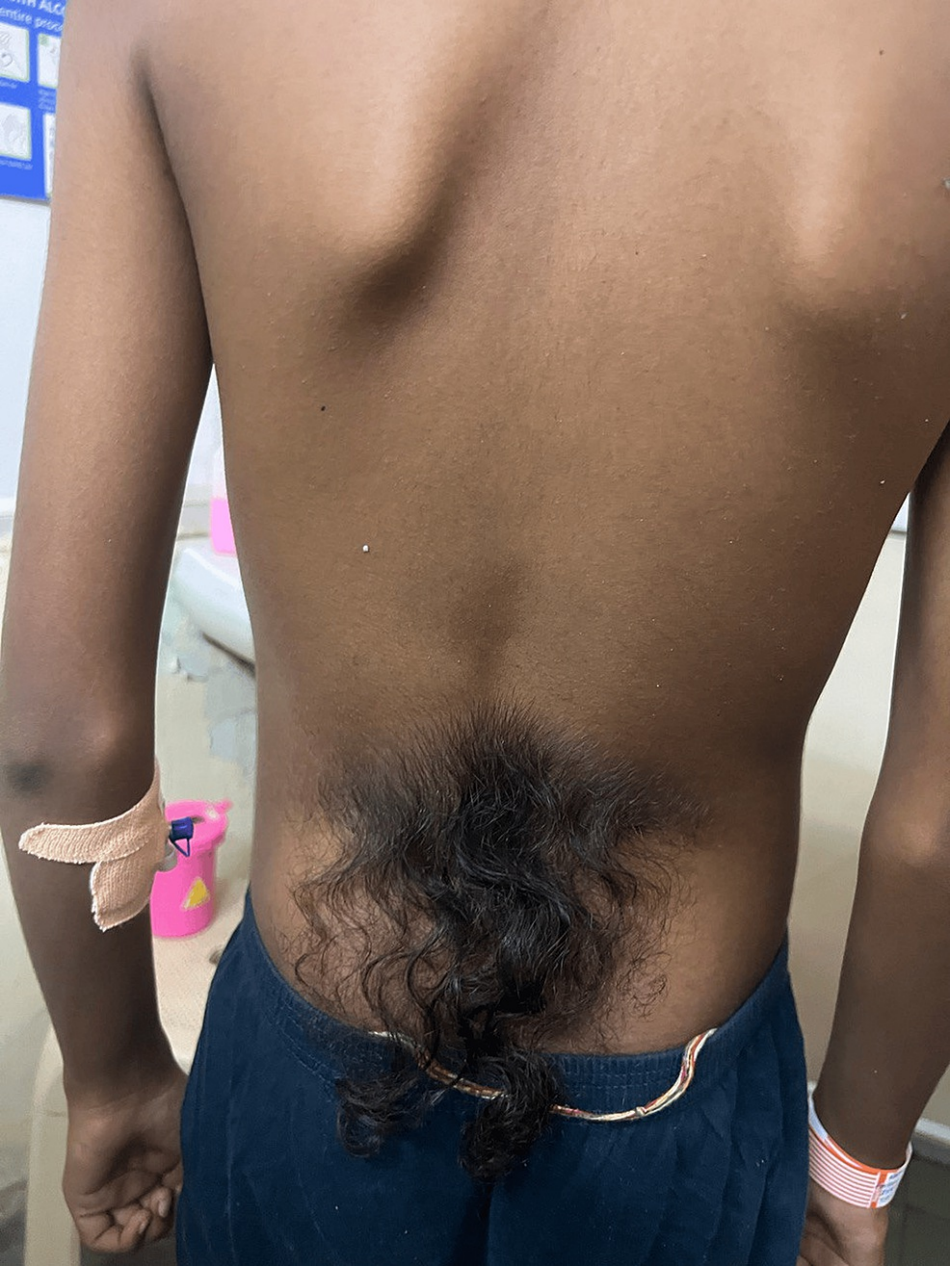
Tuft of hair on lower back of the 17-year-old male patient

On general examination, the patient was conscious and well-oriented to time, place and person. The patient was hemodynamically stable and no significant findings were found on general examination. Local examination of the lower back revealed a tuft of hair as shown in Figure 1, which on deep palpation revealed a gap present between the vertebra.

**Figure 2:**
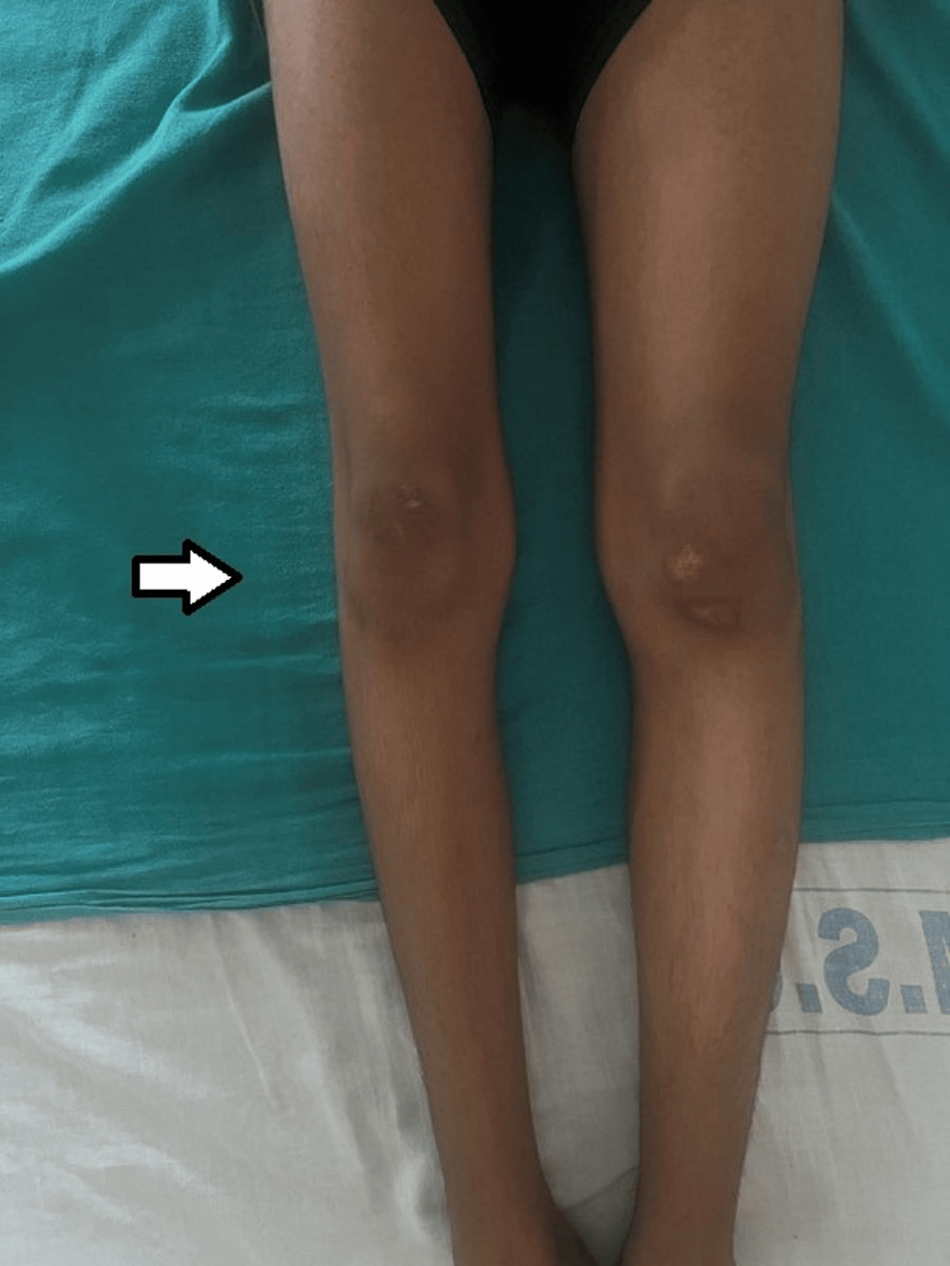
Thinning of the right lower limb as compared to the left lower limb

On detailed neurological examination, the patient’s higher mental functions were normal with no cranial nerve defects. Motor examination revealed normal bilateral upper limbs. Hypotonia was present in bilateral lower limbs. There was thinning of the right lower limb as compared to the other side as shown in Figure 2.

All deep tendon reflexes in the bilateral lower limb were absent and normal in the bilateral upper limb. Anal reflex and bulbospinous reflex were preserved. Superficial reflexes were found to be normal. Bilateral plantar reflexes were mute. Sensory examination was suggestive of decreased sensations (pain, touch, temperature, vibration) below the level of L3-L4. Sacral sensations were preserved. His neurological deficit was insidious onset, gradually progressive, asymmetric paraparesis without bowel and bladder involvement suggestive of cauda equina.

**Figure 3:**
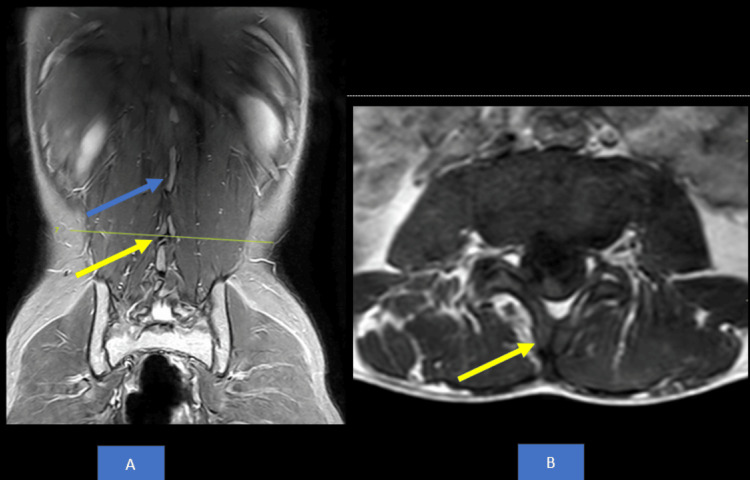
MRI of lumbosacral spine A) STIR coronal section, B) T1-weighted axial section, yellow arrow showing bifid L3 spinous process, blue arrow showing fusion of spinous process of L1 and L2 vertebra STIR: short tau inversion recovery

**Figure 4:**
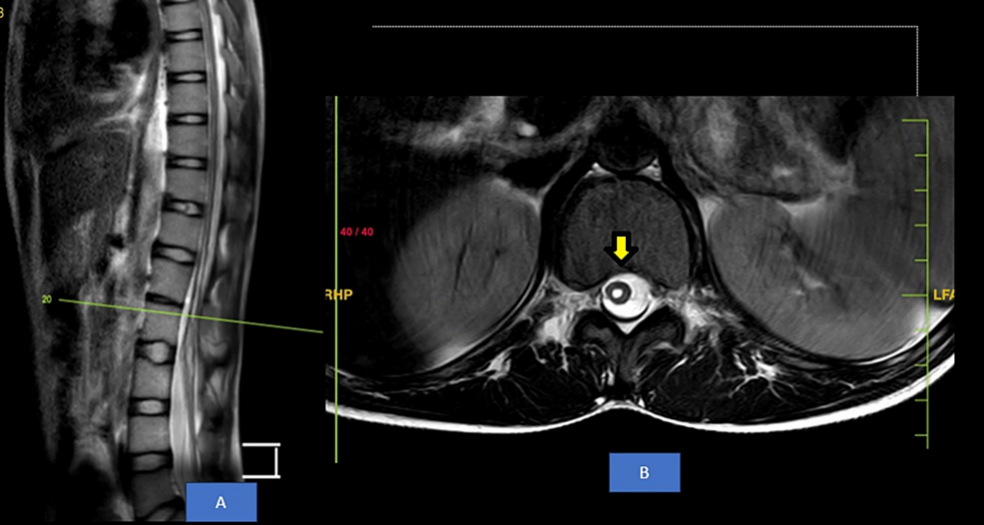
MRI Thoracolumbar Spine: A) T2-weighted sagittal section, B) T2-weighted axial section showing syrinx from T9 to L2 vertebral level (yellow arrow)

**Figure 5:**
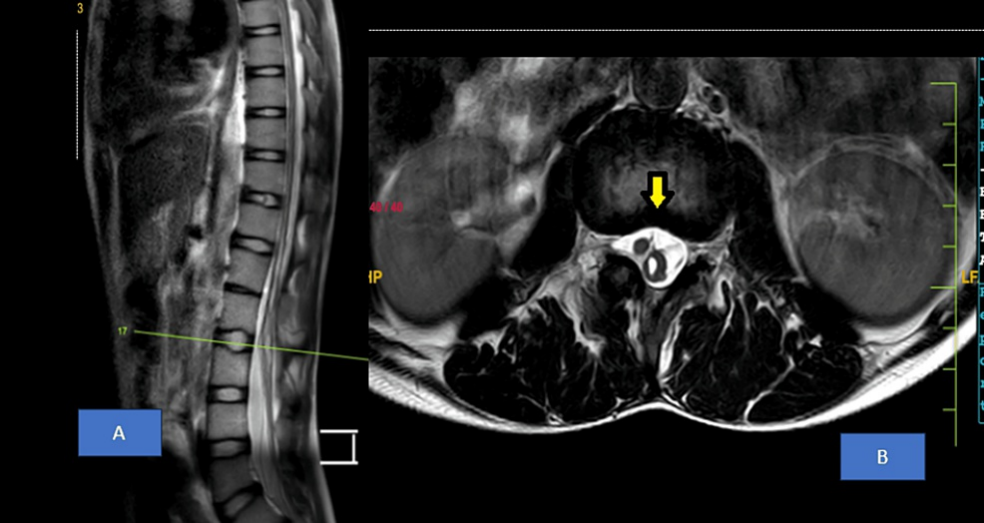
MRI Thoracolumbar Spine: A) T2-weighted sagittal section, B) T2-weighted axial section showing longitudinal splitting of the spinal cord at the level of L2 vertebra with no septa between suggestive of Type II diastematomyelia

**Figure 6:**
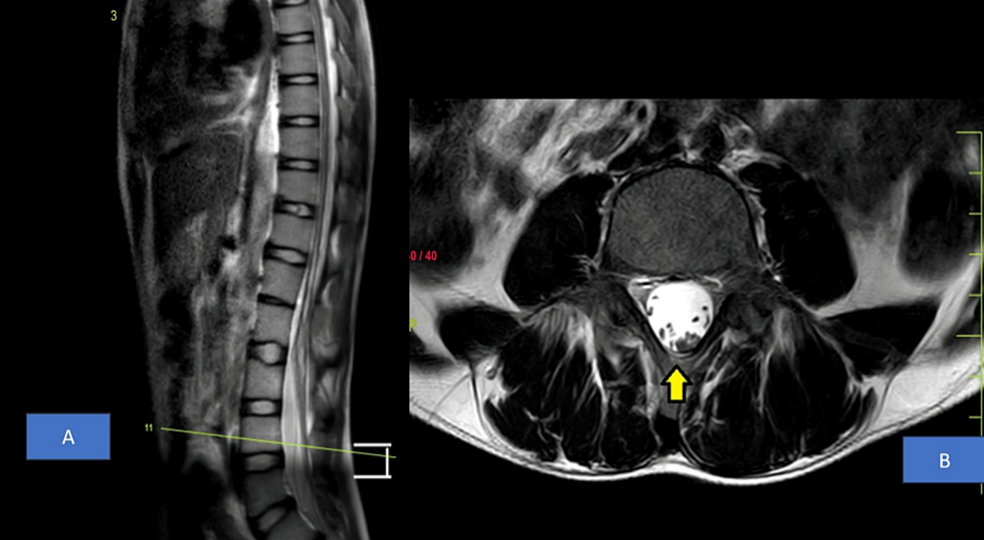
MRI Thoracolumbar Spine: A) T2-weighted sagittal section, B) T2-weighted axial section showing tethering of cauda equina nerve roots at the left lateral aspect of dural sac

MRI lumbosacral spine revealed fused spinous processes of L1-L2 vertebral bodies suggestive of partial block vertebra as shown in Figure 3. MRI Thoracolumbar Spine showed syrinx from T9 to L2 vertebral level as shown in Figure 4. There was increased spinal canal diameter at L3-L4, L4-L5 and L5-L6 disc levels with evidence of a longitudinal split in the spinal cord with no bony or fibrous septum and a single dural sac from T12-L1 up to L3-L4 disc level suggestive of Type II diastematomyelia as shown in Figure 5. The spinal cord appeared to be tethered to the ligamentum flavum at L5 lumbar level and ended at L5 lumbar vertebral level (low lying), suggestive of tethered cord syndrome (Figure 6).

Hence, we made a diagnosis of patient as complex spinal dysraphism with spina bifida occulta with Type II diastematomyelia with tethered cord syndrome.

After a tethered cord syndrome diagnosis was made, the patient was referred to the neurosurgery division for consultation, and surgery was scheduled. 

## Discussion

In this case report, we discuss a 17-year-old boy who presented with cauda equina syndrome. He had slight gait disturbance and thinning of right thigh and leg muscles which developed over the last two to three years. After radiological imaging, a diagnosis of spina bifida occulta with Type II diastematomyelia with tethered cord syndrome was made.

Spinal dysraphism is a pathologic disease associated with incorrect caudal neuropore closure. It includes every disorder connected to spina bifida. Dimples, dermal sinuses, subcutaneous lipomas, port wine stain, acrochordons, hemangiomas, aplasia cutis, telangiectasia, capillary malformation, etc. are a few other cutaneous indicators. In the third to fifth week of intrauterine life, the neuroectoderm and epithelium ectoderm begin to separate, and this process is started along the posterior midline [2].

**Figure 7:**
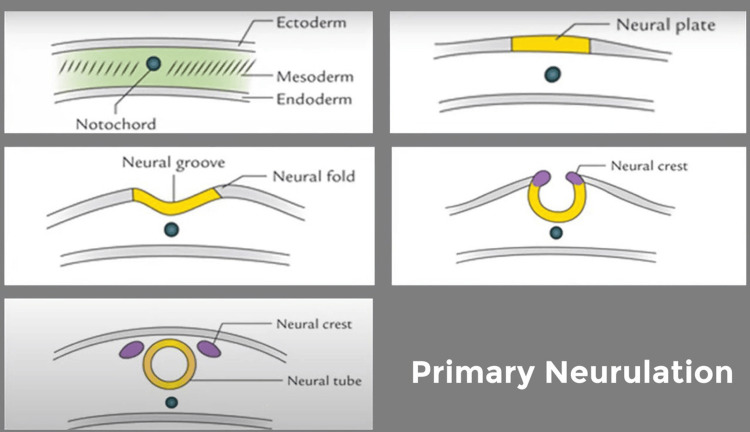
Primary neurulation Author's own creation

**Figure 8:**
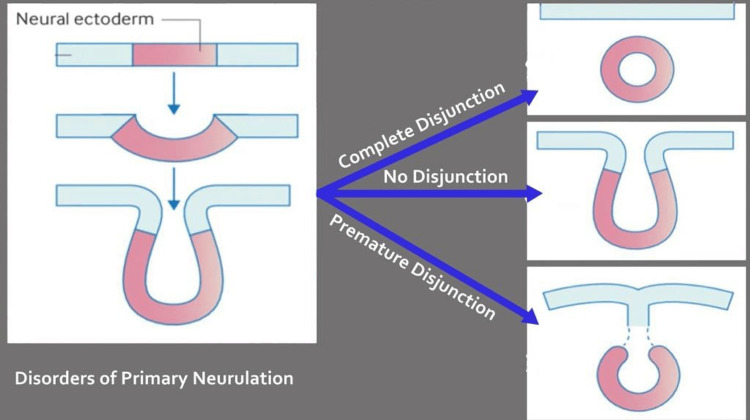
Disorders of primary neurulation Author's own Creation

**Figure 9:**
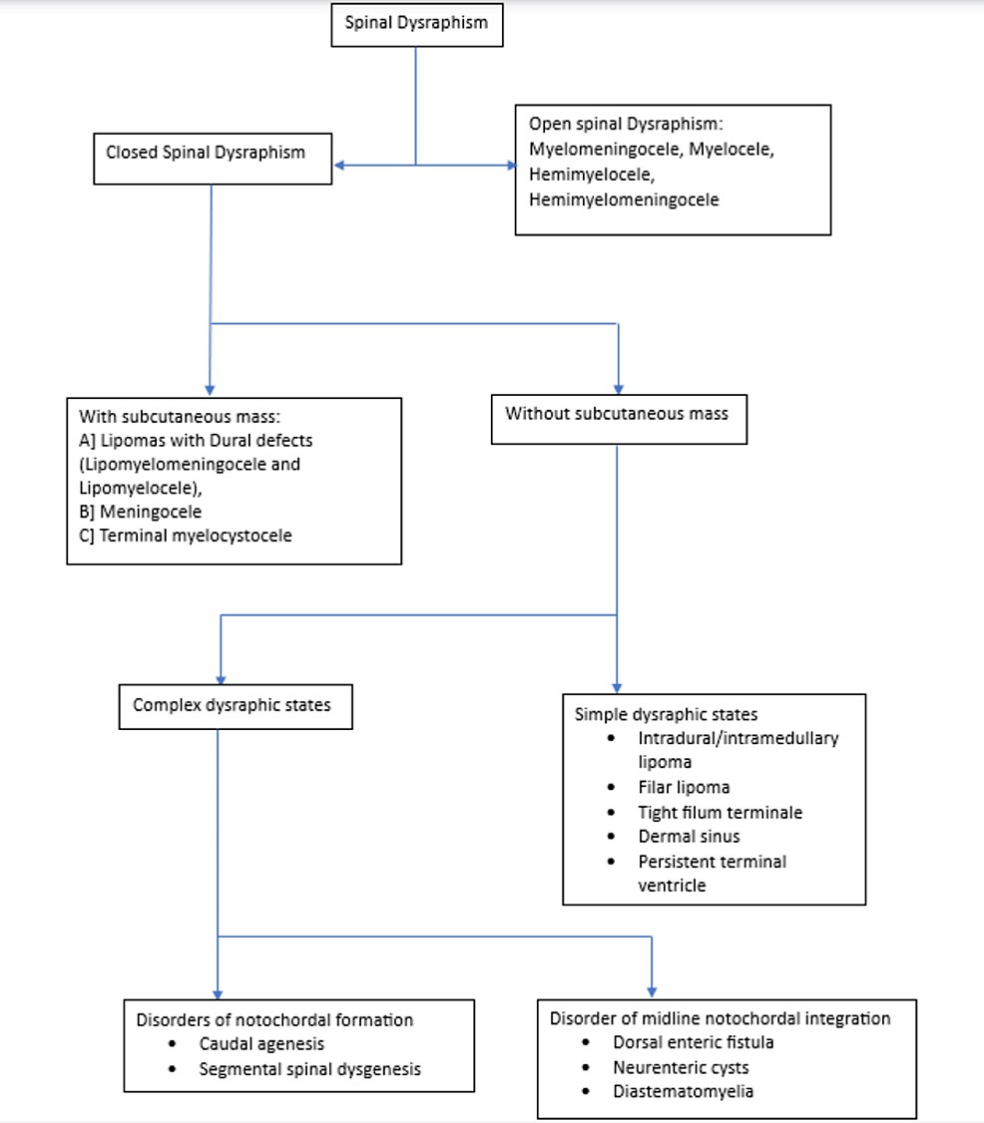
Types of spinal dysraphism Author's own creation

Understanding the pathogenesis, neuroradiologic scenarios, and clinical-radiologic classification of congenital malformations of the spinal cord requires knowledge of the three main steps (gastrulation, primary neurulation, and secondary neurulation) in the normal embryologic development of the spinal cord. With the use of a clinical examination or a neuroradiologic analysis, SDs can be categorized into two main categories: open SDs and closed SDs [2]. Primary neurulation and disorders of primary neurulation are shown in Figure 7 and Figure 8 respectively. Types of SD are shown in Figure 9.

Open spinal dysraphism (spina bifida aperta) is a gap in the spinal column that causes the meninges or the meninges and spinal cord (myelomeningocele) to herniate through the defect.

Closed spinal dysraphism (CSD), often referred to as occult spinal dysraphism or spina bifida occulta, is defined by unexposed neural tissue and improper posterior vertebral arch fusion, with intact skin covering the defect. Isolated spinal bone abnormalities make up the more prevalent and less serious types. The spinal cord malformations, such as split spinal cord malformation, or different cavitary defects of the spinal cord may coexist with the vertebral defects, making them more severe.

The clinical presentation varies to some degree by age. Younger children tend to present with cutaneous markers that lead to an evaluation for CSD. They usually do not present with neurologic symptoms. However, on formal testing, most have mild signs of lower motor neuron dysfunction and abnormalities on urodynamic testing [3]. Older children and adolescents typically show either developing neurologic impairments or cutaneous stigmata. Adults might present with enuresis as the primary complaint due to adult primary tethered cord syndrome [4].

The neurologic manifestations in patients with CSD are highly variable. Patients most often present with signs and symptoms related to lumbosacral spinal dysfunction, with autonomic and sphincteric dysfunction being more common and occurring earlier than sensorimotor deficits in the legs. Less commonly, patients can present with meningitis due to a ruptured dermal sinus or cyst. Some affected individuals are completely asymptomatic. The nature of the neurologic deficit may be static or progressive, with tethering and cord compression by extradural masses being common causes of progression [4].

Lesions that are asymptomatic in infancy and early childhood may subsequently lead to neurologic deficits if untreated, which stresses the importance of thorough evaluation and early treatment in these patients. Autonomic symptoms usually consist of urinary retention or incontinence, more subtle symptoms such as dysuria or recurrent urinary tract infections, or bowel obstruction in infancy or intractable constipation in childhood. Sensorimotor symptoms may include leg weakness, decreased or increased muscle tone, or sensory deficits of the legs and perineal area. In some cases, sensory loss can lead to atrophic ulceration of the skin.

Diastometamyelia is an uncommon congenital condition that causes the spinal cord to split longitudinally into two hemicords. Each hemicord in this anomaly has a central canal, dorsal root, and ventral root. A bony, fibrous, or cartilaginous septum that develops from the posterior surface of the anterior wall and advances dorsally toward the spinal canal is thought to be the source of diastematomyelia. Consequently, the notochord develops abnormally between the 15th and 18th day of pregnancy. The preferred diagnostic procedure for this condition is MRI [2].

Diastematomyelia has two categories. Two dural sacs surround each hemicord in kind I, the traditional kind, and a common spur (bony, cartilaginous, or fibrous) divides the spinal canal. Patients of this class frequently exhibit symptoms. A single dural sac that surrounds both hemicords and, in the majority of cases, is devoid of a septum called Type II. The majority of Type II patients exhibit no symptoms [5].

They are a diverse group that can cause life quality impairment, especially when linked to musculoskeletal, gastrointestinal, genitourinary, or respiratory system malformations [4]. They can also have mild clinical manifestations that go unnoticed or are found during a clinical examination.

Tethered cord syndrome is a result of stretch-induced malfunction of the conus and caudal spinal cord. It can manifest as a number of CSDs, including split cord malformations, tight filum terminale, spinal lipomas, and caudal regression syndrome. Back pain, bladder issues, leg weakness, calf muscle atrophy, decreased or absent deep tendon reflexes, and dermatomal sensory loss are among the constellation of symptoms that are variously linked to tethered cord syndrome. Progressive scoliosis and other foot abnormalities are examples of orthopaedic symptoms [4].

Tethered cord syndrome often manifests in toddlers and kids as progressive motor and sensory dysfunction, which may include aberrant walking patterns and loss of bladder control. Leg, perineum, and lumbosacral discomfort are more frequently reported by older kids and teenagers. The tethered cord syndrome does not account for the upper motor neuron indications and causes spinal dysfunction caudal to the T12/L1 spinal level. Therefore, if higher motor neuron symptoms (such as spasticity or hyperreflexia) are present, patients should be assessed for more proximal cord injuries [4].

After learning to walk normally, children with tethered cord syndrome start to stumble, which is the typical course of symptoms. When they are successfully trained to use the toilet, they begin dribbling urine. Later, individuals experience musculoskeletal indications and symptoms; typical observations include scoliosis, foot drop, and painless sores [6].

While younger children frequently become more irritable and refuse to engage in particular activities and motions, older children frequently complain of back discomfort that is made worse by exercise. However, there is rarely a genuine complaint of pain in the younger children. Adults with tethered cord syndrome typically experience back pain, leg discomfort, and scoliosis, and it can be challenging to identify these symptoms from other, more typical causes of chronic back pain. Ankle dorsiflexion weakness is typically the first symptom of motor impairment in older children and adults with tethered cord syndrome. Particularly when they are associated with tethered cord disease, sensory symptoms are frequently spotty and nebulous [4].

Neurogenic bladder dysfunction, whether or not associated with tethered cord syndrome, is occasionally linked to closed spinal dysraphism [7]. The real incidence of urologic involvement with CSD is unknown, and the diagnosis of bladder dysfunction is frequently postponed, especially in children who have not yet learned to use the toilet [2].

The typical accompanying clinical signs, particularly the presence of dermal stigmata, a subcutaneous mass in the back, or neurological symptoms resembling tethered cord syndrome, point to the diagnosis of CSD. A spinal dysraphic lesion can be seen on radiologic imaging, which supports the diagnosis [4].

Any phase of cleavage has the potential to be imperfect, leading to a defect in the skin, vertebrae, spinal cord, and/or central nervous system. The presence of congenital hypertrichosis over the LS region indicates the presence of spinal dysraphism [4].

Our patient had neurological deficits along with proven spina bifida, diastomatomyelia, and regional hypertrichosis. The neurosurgeon advised the patient to have the bone spur removed and the cord untethered because failing to do so could result in further neurological impairment developing in the future. He primarily complained of weakness, discomfort, and tingling in one lower limb.

Here we present the varying presentation of complex spinal dysraphism as discussed in various journals over the years. This highlights the need for evaluation of this disease based even on the cutaneous markers, irrespective of the age group. Varying presentations of complex spinal dysraphism are discussed in Table 1.

**Table 1:** Varying presentation of complex spinal dysraphisms

Authors, Journal and Year	Title	Age of Presentation/Gender	Diagnosis	Neurological Presentation
Rathore L, Sahana D, et al. [7], Neurology India/2022	Distinct Spinal Dysraphisms Arising from Each Hemicord of Type I Split Cord Malformation - A Rare Coexistence	6 months, female	Spinal dysraphisms in a split cord malformation	Paraparesis with absent deep tendon reflexes.
Gbadamosi WA, Daftari A, et al. [8] Cureus/2022	Focal Diastematomyelia in an Adult: A Case Report	22 years, female	Focal diastematomyelia	Conus medullaris of the spinal cord vertebral level L2-L3
Kaminker R, Fabry J, et al. [9] Spine Journal/2000	Split Cord Malformation With Diastematomyelia Presenting As Neurogenic Claudication in an Adult: A Case Report	Adult male	Diastometamylia at L3–L4 with spinal stenosis	Neurogenic claudication without neurologic deficit
Singh N, Singh DK, et al. [10] Journal of Pediatric Neurosciences/2015	Diastematomyelia With Hemimyelomeningocele: An Exceptional and Complex Spinal Dysraphism	3 month, female	Spinal cord malformation Type 1 with right hemimyelomeningocele	Complete flaccid paralysis of right lower extremity with normal anal tone.
Okechi H, Albright AL, et al. [11] Hindawi Journal/2012	Tethered Cord Syndrome Secondary to the Unusual Constellation of a Split Cord Malformation, Lumbar Myelomeningocele, and Coexisting Neurenteric Cyst	17 years, female	Type II split cord malformation with a large bone spur and an intradural neurenteric cyst in addition to lumbar myelomeningocele	Myelopathy and urinary incontinence
Mamo G, Batra R, et al. [3] Cureus/2021	A Case of Diastematomyelia Presenting With Minimal Neurologic Deficits in a Middle-Aged Patient	50 years, male	Diastematomyelia of the thoracic spine	Spina bifida of the lumbar region with residual paraplegia

## Conclusions

We have reported a 17-year-old patient presenting with cauda equina syndrome secondary to spina bifida occulta with tethered cord syndrome with Type II diastomatomyelia which was seen on neuroimaging. Tethered cord is a clinical syndrome associated with short and thick filum terminale. It can occur because of congenital or acquired reasons and can lead to progressive neurological deficits. A thorough medical history, physical examination, imaging, and electrophysiological tests are helpful in its diagnosis. Follow-up of patients diagnosed with spina bifida during the growth period is important to prevent complications such as syringomyelia and tethered cord syndrome.
